# A Novel Method to Identify Routes of Hepatitis C Virus Transmission

**DOI:** 10.1371/journal.pone.0086098

**Published:** 2014-01-23

**Authors:** Cyrille Féray, Julie Bouscaillou, Bruno Falissard, Mostafa K. Mohamed, Naglaa Arafa, Iman Bakr, Mostafa El-Hoseiny, Mai El Daly, Sherif El-Kafrawy, Sabine Plancoulaine, Mohamed Abdel-Hamid, Valérie Thiers, Arnaud Fontanet

**Affiliations:** 1 Inserm 955, Hopital Henri Mondor, Créteil, France; 2 Institut Pasteur, Unité d’Epidémiologie des Maladies Emergentes, Paris, France; 3 Unité INSERM 669, La maison de Solenn, Paris, France; 4 Department of Community, Environmental and Occupational Medicine, Faculty of Medicine, Ain Shams University, Cairo, Egypt; 5 National Liver Institute, Menoufia University, Shebeen El-Kom, Menufia, Egypt; 6 Viral Hepatitis Research Laboratory, National Hepatology and Tropical Medicine Research Institute, Cairo, Egypt; 7 Unité INSERM 550, Paris, France; 8 Department of Microbiology, Faculty of Medicine, Minia University, Minya, Egypt; 9 Unité INSERM 785, Centre Hépato-Biliaire, Villejuif, France; 10 Conservatoire National des Arts et Métiers, Chaire Santé et Développement, Paris, France; University of Montreal, Canada

## Abstract

**Background:**

We propose a new approach based on genetic distances among viral strains to infer about risk exposures and location of transmission at population level.

**Methods:**

We re-analysed 133 viral sequences obtained during a cross-sectional survey of 4020 subjects living in a hepatitis C virus (HCV) endemic area in 2002. A permutation test was used to analyze the correlation between matrices of genetic distances in the NS5b region of all pairwise combinations of the 133 viral strains and exposure status (jointly exposed or not) to several potential HCV risk factors.

**Results:**

Compared to subjects who did not share the same characteristics or iatrogenic exposures, the median Kimura genetic distances of viral strains were significantly smaller between brothers and sisters (0.031 versus 0.102, P<0.001), mother and child (0.044 versus 0.102, P<0.001), father and child (0.045 versus 0.102, P<0.001), or subjects exposed to periodontal treatment (0.084 versus 0.102, P = 0.02). Conversely, viral strains were more divergent between subjects exposed to blood transfusions (0.216 versus 0.102, P = 0.04) or tooth filling or extraction (0.108, versus 0.097, P = 0.05), suggesting acquisition of the virus outside of the village.

**Conclusion:**

This method provided insights on where infection took place (household, village) for several socio-demographic characteristics or iatrogenic procedures, information of great relevance for targeting prevention interventions. This method may have interesting applications for virologists and epidemiologists studying transmission networks in health-care facilities or among intravenous drug users.

## Introduction

Hepatitis C is a major health concern in Egypt [1]–[2] with more than 10% of the population being infected. Many studies have demonstrated that initial spread of the infection in this country was related to the massive parenteral anti-schistosomiasis treatment campaigns during the 1960s–1970s [3]–[5]. This was confirmed through coalescent analysis [6] which modeled the rapid spread of the epidemic from the beginning of the twentieth century. In the cohort of Zawiat Razin [4], the hepatitis C virus (HCV) prevalence in the village follows a pattern typical of Nile Delta villages in Egypt with the prevalence of HCV antibodies increasing with age, from 2.7% in those <20 years, to reach more than 30% for those above 45 years. Using oral questionnaire, HCV infections could be attributed to iatrogenic factors in subjects older than 20 years, but not in younger subjects. We recently published an analysis of the same data taking into account the clustering of infections by families [7]. Based on HCV antibody status, there was highly significant evidence for intra-familial clustering of HCV infections, especially among father-offspring, mother-offspring, and sib-sib pairs.

However, how much of this clustering is related to shared at risk behaviours, predisposing genetic factors [8] or intra-household transmission could not be studied based on serological data alone.

To overcome this limitation, we developed a new approach for analyzing viral sequencing data at the household and community level. The basic hypothesis is that highly similar strains reflect either a direct transmission event or exposure to a shared source, whereas divergent strains suggest separate acquisition of HCV in time and place. To explore this hypothesis, we computed correlation coefficients relating genetic distances and exposure to a given risk factor for all pairwise combinations of subjects with viral sequences available. We used a permutation method for significance testing, the Mantel’s test [9] as it does not require independence among observations.

## Materials and Methods

### Population

A large epidemiological study was conducted between May and November 2002 in Zawiat Razin, a village of around 20,000 inhabitants in Menofia Governorate (Nile Delta). Practical details have been described elsewhere [4]. After informed consent was obtained (from the head of household for children less than 18 years old), all residents older than three years of age and living in one sector of the village (25% of the total village population) were invited to participate. A questionnaire was administered on socio-demographic characteristics (age, sex, duration of residence in the village, marital status, educational level) and iatrogenic exposures (hospitalisation, injections, dentistry, surgical and obstetrical procedures, blood transfusions) possibly associated with the transmission of HCV in the village. A second-round follow-up of the cohort was conducted between June 2003 and March 2004 [7]. First-degree relationships (sib-sib, mother-child, father-child),and spouse relationships living in the same household were recorded. Other persons living in the same household were considered as not family-related for the purpose of this study. The starting population was 4020 individuals. As previously reported [4], 475 (11.8%) were positive for anti-HCV antibodies, 456 could be tested for HCV RNA and 273/456 (60%) were serum HCV RNA positive. Because this study was interested in household transmission, we selected for sequencing viremic patients only when someone else in the household was viremic leading to a subgroup of 133 subjects.

All participants (or head of the household for children aged less than 18 years old) signed an informed consent. The study protocol was reviewed and approved by the committee for biomedical research at Institut Pasteur, Paris, and by the Institutional Review Board of the National Hepatology & Tropical Medicine Research Institute, Cairo.

### Methods

#### Amplification and sequencing

Partial amplification of the NS5B (nucleotide position 7915–8303 330 bp) was performed as described elsewhere [10]. Molecular characterization was first carried out by sequence analysis and phylogenetic studies of HCV NS5B region. A partial amplification of E1 region (nucleotide position 482–1320, 830 bp was also performed as described previously [11] in a subset of positive samples. Positive samples were purified, and bidirectional sequence analysis was performed. Consensus nucleotide sequences were used for phylogenetic studies.

#### Phylogenetic analysis

The NS5B or E1 sequences were added to a set of representative HCV genotypes and subtypes for the same gene region available in the databanks (n>100). The DNA alignments were generated with Clustal W and followed by further phylogenetic analysis using the Neighbor Joining program using 1,000 bootstrap replication with in the Mega package [12]. Positions for which an ambiguity was observed were considered as missing for the calculation of nucleotidic distances.

#### Correlation matrix analysis

Traditional statistics cannot be applied since genetic distances are pairwise defined and these distances are thus not independent from each other. Mantel's test [9] is a generalized regression permutation procedure classically used to compare two distance matrices. In our approach we correlated the genetic distance matrix consisting of pairwise Kimura two-parameter distances (d) with phenotypic matrix. They were calculated between each pair of subjects for a set of given risk factors and arranged in dissimilarity matrices where the distance was 0 if both subjects were exposed and otherwise 1 (Figure S1 in supplemental data). The studied variables were those listed in Table 1.

**Table 1:** Number of participants exposed to each risk factor.

Possible risk factor	n
Born in the village	122
Infected brother/sister	41
Infected mother	22
Infected father	15
Infected spouse	28
Infected non familial household member	62
Surgery	51
Stitches	64
Abcess drainage	33
Intravenous injections	18
Anti-schistomiasis treatment	21
Periodontal treatment	21
Tooth filling or extraction	68
Urinary catheter	11
Blood transfusion	8
Instrumental delivery	7

doi:10.1371/journal.pone.0086098.t001

The correlation between each distance matrix and each phenotypic matrix was evaluated using the Spearman correlation coefficient (R0), which ranges from –1.0 for a perfect negative to 1.0 for a perfect positive correlation between two matrices. As variables arranged into matrices are not independent (e.g. the distance between case 1 and 3 is not independent of the distance between case 1 and 2, because case 1 is involved in both), the significance of the correlation is determined by a permutation test. The rows and columns of one matrix were randomly permuted 5000 times and the Spearman correlation was calculated for each permutation. The measure of significance is given by the ratio N/5000, where N is the number of times that R0 is exceeded by correlation coefficients calculated with permuted matrices. If the original matrices are correlated, the disruption caused by the permutations should produce correlation coefficients below R0.

Analyses were conducted using STATA 11.0 software (Stata Corporation, College Station, Texas. USA).

## Results

### Study Population

The mean age of the subgroup with sequencing data was 36 years (±15; range 9–71 years) with 77 (58%) subjects less than 41 years. Seventy-eight (59%) were male. 122 (92%) were born in the village. These 133 subjects lived in 54 different houses; 38 of them had no familial relationship (spouse, child, parent, brother/sister) with any of the other included subjects. Among the others, we could analyze viral distances in 14 married couples, in 22 mother-child pairs, in 15 father-child pairs and 28 sib-sib pairs (Note: The same individual can belong to more than one pair, e.g., a child may be in a mother-child, father-child, and sib-sib pairs simultaneously).

Other risk factors for this population are described in Table 1. We only considered risk factors for which the percentage of exposed ranged between 5% and 95%, so that genetic distances estimates for each group (jointly exposed or not) could be based on sufficient numbers. Of note, many patients were exposed to more than one risk factor.

### Phylogenetic Analysis

Comparison with published reference sequences from Genbank showed that 130 (98%) of the Egyptian sequences clustered with genotype 4, and that 3 (2%) clustered with genotype 1. Most cases (105 out of 133; 77%) clustered with the subtype 4a Egyptian reference sequence ED43. The remaining sequences formed 5 distinct groups with strong bootstrap support. Two of these groups clustered with previously described Egyptian sequences identified as subtypes 4m and 4o (n = 2 and 3, respectively),and one with an undescribed yet subtype (provisionally called subtype 4u; n = 20). The last group (n = 3) clustered as a monophyletic group (bootstrap value 99%) corresponding to genotype 1g (n = 2) and 1b (n = 1). The NS5B phylogenetic classification of the Egyptian isolates was confirmed by partial amplification of the E1 region (position 482–1320) in 66 subjects. Altogether these data indicate the presence of a new subtype of type 4. Sequence access numbers are EF694393–EF694525 for the NS5B region and EF694321–EF694386 for the E1 region.

**Figure 1:**
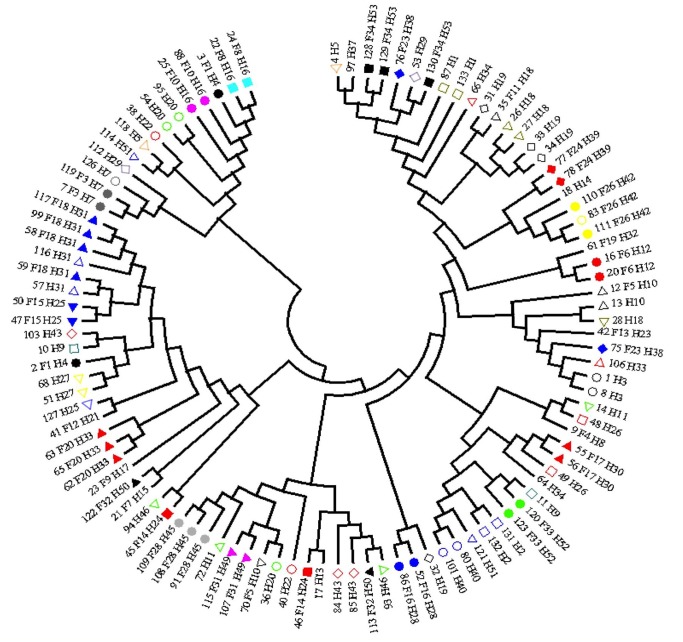
Phylogenetic tree from the HCV sequences of 105 patients infected by the genotype 4a. Same shape filled with same color means same house and same family. Same shape empty means same house, but not the same family. Absence of symbol means no connection to other participant with sequence.

Figure 1 shows the phylogenetic tree corresponding to the 105 subjects infected by HCV genotype 4a. The proximity of strains according to families and houses is suggested although bootstrap value for each familial or household cluster was always below 50%.

### Matrix Correlation

From the 133 subjects, we constructed a genetic matrix of 8778 combinations of genetic distances defined in the NS5B region and 18 corresponding phenotypic matrices of the same size.

The genetic (Kimura) distances between the strains infecting two subjects having a sib-sib, mother-child, father–child or spouse relationship, living in the same house without familial relationship, or submitted to periodontal treatment were lower than the distances between the strains infecting two subjects who did not share the same relationships (Table 2).

**Table 2:** Pairwise NS5b HCV genetic distances among 133 subjects according to common exposure patterns.

	Yes	No	rho	p
	1^st^ quart. - Median - 3^rd^ quart.	1^st^ quart. - Median - 3^rd^ quart.		
**Household/Family ties**				
Same house, no family tie	0.049 - 0.078 - 0.112	0.081 - 0.102 - 0.214	0.04	<0.001
Sib-sib	0.019 - 0.031 - 0.052	0.081 - 0.102 - 0.214	0.07	<0.001
Mother-child	0.026 - 0.044 - 0.096	0.081 - 0.102 - 0.214	0.05	<0.001
Father-child	0.032 - 0.045 - 0.052	0.081 - 0.102 - 0.214	0.05	<0.001
Spouses	0.032 - 0.061 - 0.209	0.081 - 0.102 - 0.214	0.02	0.03
Entire life in the village	0.081 - 0.101 - 0.212	0.082 - 0.154 - 0.221	0.05	0.21
**Iatrogenic exposures**				
Surgery	0.083 - 0.105 - 0.220	0.081 - 0.102 - 0.213	−0.04	0.21
Stitches	0.083 - 0.110 - 0.229	0.080 - 0.101 - 0.206	−0.09	0.05
Blood transfusion	0.114 - 0.216 - 0.467	0.081 - 0.102 - 0.214	−0.04	0.04
Bladder catheterization	0.094 - 0.106 - 0.122	0.081 - 0.102 - 0.214	−0	0.46
IV cannula	0.083 - 0.101 - 0.205	0.081 - 0.102 - 0.217	0.01	0.38
10+ injections	0.080 - 0.099 - 0.207	0.083 - 0.106 - 0.222	0.06	0.16
Treatment for abcess	0.077 - 0.095 - 0.211	0.083 - 0.102 - 0.214	0.03	0.24
Periodontal treatment	0.073 - 0.084 - 0.102	0.083 - 0.102 - 0.215	0.07	0.02
Tooth filling or extraction	0.084 - 0.108 - 0.219	0.079 - 0.097 - 0.208	−0.11	0.05
Anti-schistomiasis treatment	0.080 - 0.113 - 0.195	0.081 - 0.102 - 0.215	0.01	0.36
Obstetrical history*				
5+ delivery	0.087 - 0.106 - 0.233	0.083 - 0.102 - 0.218	−0.05	0.25
Traumatic delivery	0.076 - 0.109 - 0.113	0.083 - 0.102 - 0.218	0.03	0.29
Abortion	0.094 - 0.207 - 0.237	0.083 - 0.101 - 0.216	−0.11	0.07

doi:10.1371/journal.pone.0086098.t002

Conversely, the genetic distances between strains infecting two subjects who have been exposed to stitching, blood transfusion, or tooth filling or extraction were significantly higher than in those not sharing these risk factors (the difference was marginally significant for abortion).

We did not evidence a relationship between genetic distance and a common exposure to antischistomiasis therapy, injections, instrumental deliveries or the other studied risk factors.

**Figure 2:**
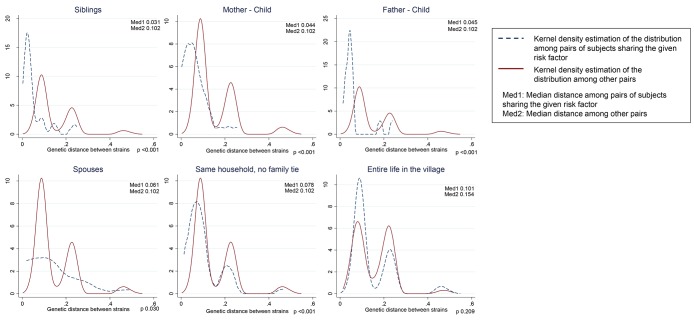
Distributions of viral genetic distances depending on household/family ties (n = 133). Among non-exposed (continuous line), the three peaks correspond to genetic distance among individuals belonging to the same subtype (left), to the same genotype, but not the same subtype (middle), and to different genotypes (right).

**Figure 3:**
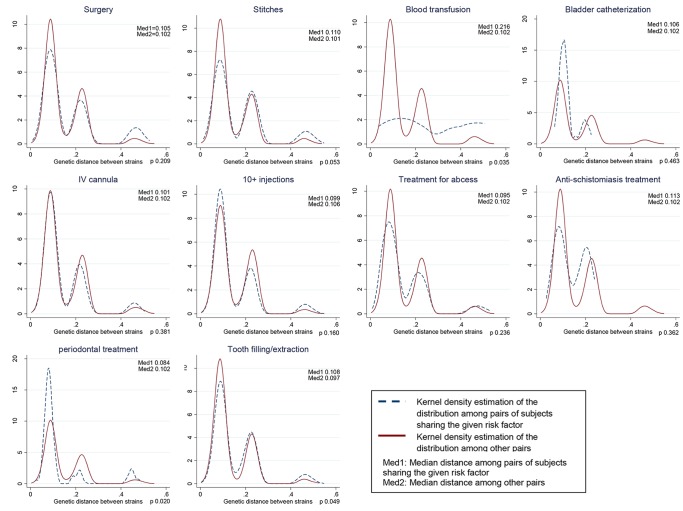
Distributions of viral genetic distances depending on iatrogenic exposure (n = 133). Same interpretation as for Figure 2.

**Figure 4:**
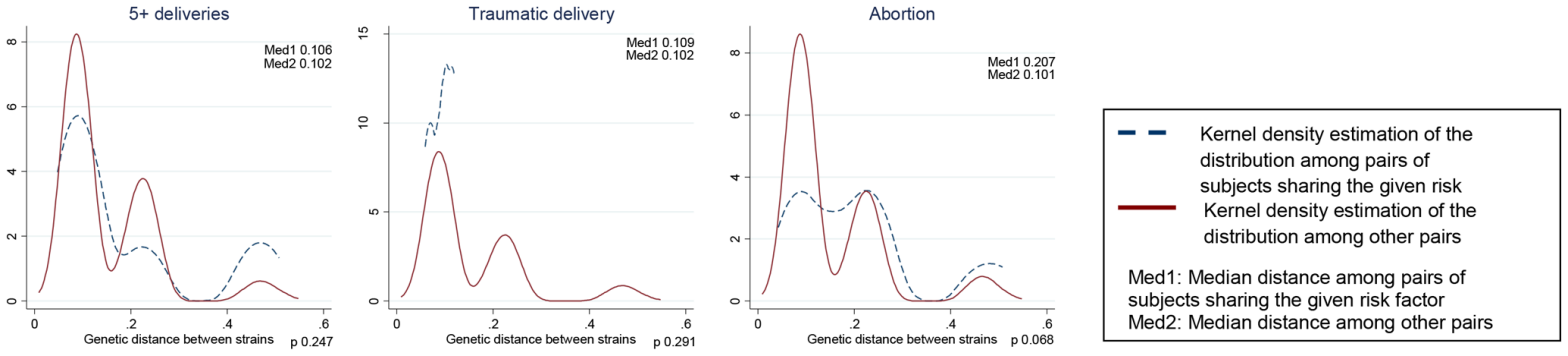
Distributions of viral genetic distances depending on obstetric risk factors (n = 55). Same interpretation as for Figure 2.

The distribution of viral genetic distances according to the risk factors is shown in the figures 2 to 4. Among non-exposed (continuous line), the three peaks correspond to genetic distance among individuals belonging to the same subtype (left), to the same genotype, but not the same subtype (middle), and to different genotypes (right). The same applies to the peaks observed among exposed (dashed lines), but low numbers may affect the visibility of the peaks. Genetic relatedness of strains translated into shifting to the left of the distribution due to lower distances in the first peak (within subtype) among exposed compared to non-exposed (e.g., siblings). Genetic remoteness of the strains translated into shifting to the right of the distribution due to a higher right peak following the introduction of a new genotype, genotype 1, in the village (e.g., blood transfusion).

The same analysis was repeated using genetic distances derived from the E1 region of 66 subjects. There was a high correlation between the pairwise genetic distances calculated from NS5b and E1 regions (Fig. S1). The risk factor analysis using the E1 region pairwise distances found the same results as with the NS5b region, except for non-related household members and spouses, which no longer showed the same level of closeness, and for stitches which were no longer more distant (Table 3). The same analysis was performed in the group of 105 subjects with infection by genotype 4a and the same relationships were evidenced in this group (data not shown).

**Table 3:** Pairwise E1 HCV genetic distances among 66 subjects according to common exposure patterns.

	Yes	No	rho	p
	1^st^ quart. - Median - 3^rd^ quart.	1^st^ quart. - Median - 3^rd^ quart.		
**Household/Family ties**				
Same house, no family tie	0,080 - 0,113 - 0,195	0,086 - 0,110 - 0,227	0,03	0.01
Sib-sib	0,024 - 0,049 - 0,079	0,086 - 0,110 - 0,227	0,06	0.001
Mother-child	0,026 - 0,044 - 0,210	0,086 - 0,110 - 0,227	0.02	0.05
Father-child	0,032 - 0,061 - 0,219	0,086 - 0,110 - 0,227	0,05	0.01
Spouses	0,083 - 0,105 - 0,220	0,086 - 0,110 - 0,227	0,02	0.10
Entire life in the village	0,091 - 0,170 - 0,232	0,112 - 0.190 - 0.242	0,05	0,31
**Iatrogenic exposures**				
Surgery	0,083 - 0,105 - 0,260	0,083 - 0,120 - 0,224	−0,04	0,25
Stitches	0,073 - 0,110 - 0,227	0,084 - 0,120 - 0,222	−0,1	0,1
Blood transfusion	0,114 - 0,216 - 0,467	0,079 - 0,123 - 0,214	−0,04	0,05
Bladder catheterization	0,083 - 0,105 - 0,220	0,086 - 0,110 - 0,227	0	0,46
IV cannula	0,087 - 0,110 - 0,233	0,086 - 0,110 - 0,227	0,009	0,58
10+ injections	0,080 - 0,106 - 0,259	0,086 - 0,110 - 0,227	0	0,21
Treatment for abcess	0,081 - 0,106 - 0,253	0,086 - 0,110 - 0,227	0,02	0,34
Periodontal treatment	0,080 - 0,102 - 0,228	0,086 - 0,110 - 0,227	0,005	0,09
Tooth filling or extraction	0,084 - 0,108 - 0,219	0,071 - 0,090 - 0,217	−0,15	0,07
Anti-schistomiasis treatment	0,087 - 0,103 - 0,233	0,081 - 0,102 - 0,215	0,02	0,36
Obstetrical history*				
5+ delivery	0,083 - 0,156 - 0,267	0,087 - 0,106 - 0,243	−0,06	0,34
Traumatic delivery	0,061 - 0,102 - 0,209	0,087 - 0,106 - 0,239	0,02	0,37
Abortion	0,091 - 0,212 - 0,243	0,087 - 0,105 - 0,233	−0,1	0,17

doi:10.1371/journal.pone.0086098.t003

## Discussion

The proposed approach is an original application of the matrix correlation test to the issue of viral transmission among a limited population of infected subjects exposed to multiple risk factors. It allows identification of risk factors that would, in a given community, lead to either homogeneous strains (when transmission occurs among members of that community or through exposure to a common source of infection), or divergent strains (when infections are contracted outside of the community or at different times). We demonstrated that two relatives living in the same house were infected by less divergent strains than two subjects living in different houses or belonging to different families. Also, two subjects having been exposed to periodontal treatment were infected by more similar strains compared to others. Conversely, two subjects exposed to blood transfusions, or tooth filling or extraction had more divergent strains than others.

The approach was particularly powerful to document intrafamilial transmission of HCV (i.e., transmission of HCV among two members of the same family). Such demonstration has proven to be difficult in the past with classical epidemiological studies (reviewed in 13). Many studies relied on clustering of HCV infections based on positive serology only. In such circumstances, one cannot exclude common at risk behaviours shared among members of the same family. More convincing are studies using sequences homologies to document intrahousehold transmission. Such studies were few, and with limited statistical power. In that same village, it took us four years of follow-up to identify 25 seroconverters among 3184 subjects. Of these, 17 were viremic, and 2 only had another family member infected with the same strain indicating intrafamilial transmission 14. With the new approach described in this paper, we obtained similar evidence for intrahousehold transmission with a much smaller number of individuals and a cross-sectional survey only. Our approach, based on sampling of households with multiple cases, is quick to realize (cross-sectional) and pose no methodological challenges (no need for complex strategies in choosing the controls of case-control studies).

HCV is classically considered as poorly contagious within families or between spouses 15–19. In western countries, clear parenteral routes (intravenous drug usage and transfusions before 1991), are the main routes of transmission. However, subjects with no identifiable route of infection represent 10% to 20% of all cases in most Western countries series 20–21 and were frequent in the studied Egyptian subjects, particularly among children 4. Our results indicate that an important source of contamination comes from the household and is particularly strong among siblings and between parents and children pointing these relationships as targets for screening and prevention. Further behavioural studies are needed to clearly identify the route of infection which may involve either classical modes of transmission (e.g., shared medical injections) or minute amounts of blood, or other body fluids such as saliva. Transmission between spouses was likely in this study and others performed in other rural communities of Egypt 22, but remains an issue of debate 17–19.

Other risk factors identified through this analysis were periodontal treatment, tooth filling or extraction, and blood transfusion. All, except for tooth filling or extraction, were risk factors for HCV infection in previous reports from the same community 4,7, with the additional information provided here that periodontal treatment was most likely performed within the village since subjects reporting this practice had more similar strains, while tooth filling or extraction, and blood transfusions were performed outside of the village since subjects reporting these practices had more divergent strains. This information is particularly useful for public health practitioners when trying to identify where infection takes place in order to propose corrective measures. In this context, it would be useful to examine what type of periodontal treatment is performed within the village, and how the material is cleaned between patients. Local practices include cleaning of gums by non medical staff with pieces of metal for aesthetic purposes, and may be associated with HCV transmission if not properly sterilised between individuals. Of note, periodontal treatment has also been reported associated with HCV infection in a case-control study analyzing incident infection in Cairo 23. Bleeding during periodontal treatment is frequent. A study reported the detection of HCV RNA on instruments after dental treatment in HCV infected patients 24.

This approach should not be used as a replacement of classical epidemiological studies, such as case-control or cohort studies, for the identification of risk factors. Indeed, it will not identify procedures taking place both within and outside of the community, as shown here by the lack of significant relationships for risk factors such as treatment for schistosomiasis, intravenous injections, or obstetrical risks. The fact that history of treatment for schistosomiasis did not appear as a risk factor in our analysis may also be related to the very old timing of this exposure which took place 30 to 40 years prior to this survey. Although the strains that spread during the mass treatment campaigns might have been closely related initially, they had time to diverge since, and cannot be recognized during this analysis. Conversely, the approach may be biased towards emphasizing intrafamilial transmission if strains infecting two siblings experience lower divergence over time due to similar HLA-driven immune response targeting epitopic sites.

This same Mantel test has been successfully used in different fields with the purpose of comparing matrix variables 25–26. It has also been applied in the field of virology for the study of cellular tropism of HIV 27–28 or HCV quasispecies 29–30 or for the study of relations between variability of genotype 1b and the severity of fibrosis after liver transplantation 31. It has been used to explain spatial patterns in genetic distances among different populations of human, animals 32 or bacteria. However it has never been applied to the issue of viral transmission.

In conclusion, this study has shown an interesting application of a new statistical approach to the analysis of viral genetic distances to better understand transmission routes of infection. It has clearly documented the distinct participation of intrafamilial and intrahousehold components in the transmission of HCV in a rural area of Egypt. It has also pointed at periodontal treatment as a potential source of HCV transmission within the village. Other applications of this technique may be envisaged for the investigation of transmission networks in health care facilities or among intravenous drug users.

## Supporting Information

Figure S1 **Scatterplot of correlation between the pairwise genetic distances derived from NS5b and E1 regions in a subsample of 66 participants.**
(TIF)
