# Supplementary figures and images for: A Novel Method to Identify Routes of Hepatitis C Virus Transmission
Source: PLoS One. 2014 Jan 23;9(1):e86098. doi: 10.1371/journal.pone.0086098 (PMC3900465; doi:10.1371/journal.pone.0086098)

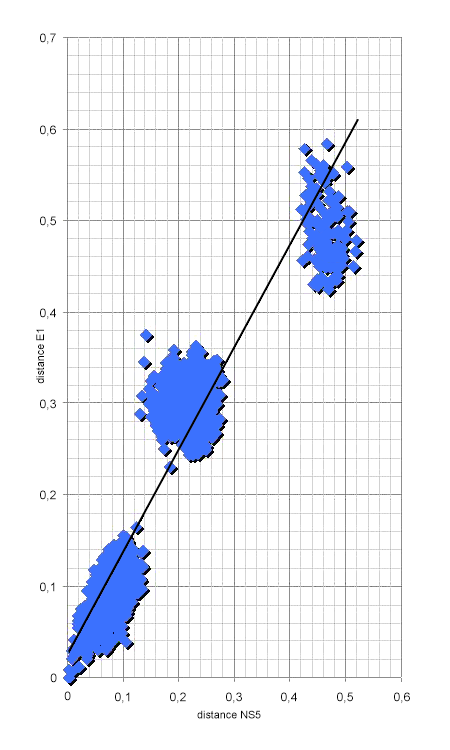

Supplement: Figure S1 — (TIF) [file pone.0086098.s001.tif]
